# Self-Assembled Nano-Fe_3_C Embedded in Reduced Graphene Oxide Aerogel with Efficient Fenton-Like Catalysis

**DOI:** 10.3390/nano10122348

**Published:** 2020-11-26

**Authors:** Liping Wang, Mingyu Zhang, Jiawei Xie

**Affiliations:** 1 College of Biological and Environmental Engineering, Changsha University, Changsha 410022, China; xiejiawei199708@163.com; 2 State Key Laboratory for Powder Metallurgy, Central South University, Changsha 410083, China

**Keywords:** self-assembled nano-Fe_3_C@RGO aerogel, hydrothermal synthesis, high temperature treatment, Fenton-like, methyl orange

## Abstract

Aiming at the removal of refractory organic pollutants in aqueous solution, self-assembled nano-Fe_3_C embedded in reduced graphene oxide (nano-Fe_3_C@RGO) aerogel was prepared by hydrothermal synthesis and high temperature treatment, and characterized by SEM, HRTEM, pore size distribution, XRD, XPS and FTIR. The results showed that the aerogel was porous, and most of the Fe_3_C particles were less than 100 nm in size. They were evenly dispersed and embedded in the RGO aerogel. Furthermore, the mapping images confirmed that the elements of carbon, nitrogen and iron were homogeneously distributed. Moreover, the specific surface area of the aerogel was up to 324.770 m^2^/g and most of the pore sizes were between 5 and 10 nm. The formation of nano-Fe_3_C was identified by XRD pattern and HRTEM. Analysis of an XPS spectrum indicates that the nano-Fe_3_C was embedded in the graphene layer. The aerogel contained a large number of functional groups, including –NH_2_, –NH and –C=O, etc., which greatly strengthened the adsorption of organics. Finally, the Fenton-like catalytic degradation properties of the self-assembled nano-Fe_3_C@RGO aerogel were investigated by testing the removal of methyl orange from the aqueous solution. The results showed that the value of *C_t_*/*C*_0_ decreased to 0.050 after 240 min, suggesting a high degradation rate was obtained. Meanwhile, the chemical reaction was verified in accordance with the first-order kinetic model, and the higher temperature was beneficial to the catalytic degradation. At the same time, methyl orange was degraded into small molecules by the hydroxyl and superoxide radicals generated during the reactions. Therefore, the self-assembled nano-Fe_3_C@RGO aerogel, as a novel Fenton-like catalyst, introduces a new approach in the field of treatment of refractory organic wastewater.

## 1. Introduction

In recent years, the Fenton process has become a promising wastewater treatment technology because of its strong oxidative ability and environmentally friendly nature. During the Fenton reaction, H_2_O_2_ is decomposed into hydroxyl radicals (HO·) under the catalysis of Fe^2+^ and then, the hydroxyl radicals are prone to react with organic pollutants. The Fenton reaction can decompose organic matter into small molecules by means of electron transfer. Unfortunately, the traditional Fenton technique gives rise to a loss of catalytically active ions and produces a lot of sludge. Therefore, Fenton-like technology is emerging to overcome the shortcomings of traditional Fenton technology [1,2,3,4,5,6,7,8].

The key to Fenton-like technology is the screening of carriers and the preparation of the supported catalyst. High specific surface area and corrosion resistance are required to catalyze carriers in Fenton-like technology. The RGO has a large specific surface area, excellent electron transfer capacity, and a pi-delocalized structure similar to that of benzene-like aromatic nucleus, which has good adsorption performance for organic compounds with aromatic nucleus. Therefore, RGO and its complexes have received extensive attention in the treatment of organic wastewater by Fenton-like technology [9,10,11]. However, two-dimensional RGO is easy to overlap, leading to a reduced specific surface area; thus, its capacity to support the catalyst is decreased. Moreover, Fenton-like catalysts are mainly iron-based catalysts such as nanometer zero-valent iron [7,12,13,14,15], Fe_2_O_3_ [4,16,17,18], Fe_3_O_4_ [19,20,21,22,23,24], FeOOH [10,25,26,27,28,29,30], etc. Some of these catalysts are easy to oxidize, while others are easy to aggregate in the preparation process.

Herein, we proposed a self-assembled nano-Fe_3_C@RGO aerogel for the removal of refractory organic pollutants in aqueous solution. The RGO aerogel is a three-dimensional structure with a high specific surface area and many active sites, thus the RGO aerogel can easily support a large number of catalysts. Furthermore, compared with other iron-based catalysts, Fe_3_C has the advantages of not oxidizing easily, eco-friendliness, and difficult agglomeration [31,32]. Therefore, the self-assembled nano-Fe_3_C@RGO aerogel was prepared by hydrothermal synthesis and high-temperature treatment, and its Fenton-like catalysis properties and mechanisms were investigated in detail.

## 2. Materials and Methods

### 2.1. Experimental Materials

The graphene oxide solution was purchased from Chengdu Institute of Organic Chemistry, Chinese Academy of Sciences (Chengdu, China). Iron nitrate, ammonia, methyl orange and β cyclodextrin were all used as analytical reagents (AR) produced by China Pharmaceutical Group Co. LTD (Beijing, China) and 30% hydrogen peroxide (AR) was produced by Hunan Huihong Co. LTD (Changsha, China).

### 2.2. Preparation of Self-Assembled Nano-Fe_3_C@RGO Aerogel

**Figure 1:**
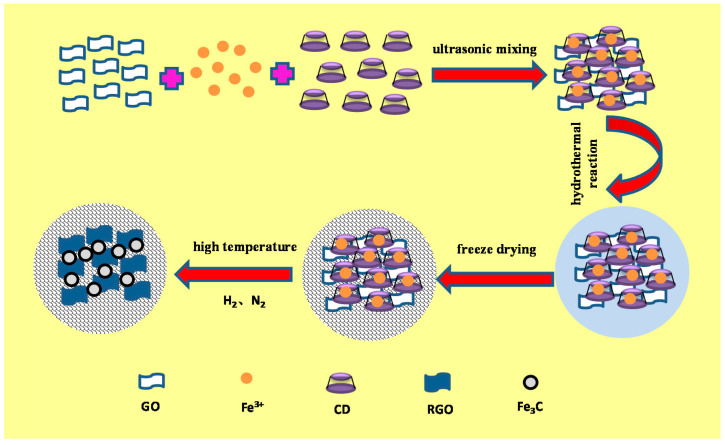
The formation mechanism of self-assembled nano-Fe_3_C@RGO aerogel.

Firstly, the graphene oxide solution was diluted to 5 mg/mL using deionized water and sonicated in an ultrasonic cleaner at 30 °C for 30 min. Then, the pH of the solution was adjusted to 10 by adding ammonia and used consecutively for ultrasonic shaking at 30 °C for 1 h. Afterwards, the solution was ultrasonically stirred for 1 h at 30 °C after adding 10 mL of 0.02 mol/L ferric nitrate solution. Following dropwise addition of 10 mL of 30 mg/mL β cyclodextrin solution as a crosslinking agent, ultrasonic agitation was employed at 30 °C for 2 h. After that, the mixture solution was put into a hydrothermal reactor to execute a hydrothermal reaction at 150 °C for 6 h. Next, the hydrogel was taken out and washed with deionized water 3 times to remove the unreacted substances on the surface. Then, a vacuum freeze dryer was used to freeze and dry the hydrogel for 48 h to remove moisture. Finally, the self-assembled nano-Fe_3_C@RGO aerogel was prepared at 650 °C in a tube furnace, with hydrogen and nitrogen injected simultaneously for 2 h. Its formation mechanism is described in Figure 1.

### 2.3. Characterization

The morphology of the self-assembled nano-Fe_3_C@RGO aerogel was observed using a scanning electron microscope (SEM, Zeiss Sigma HD, Oberkochen, Germany). Transmission electron microscopy (TEM, JEOL JEM-2010, Tokyo, Japan) was employed to investigate the structure and obtain detailed information about the self-assembled nano-Fe_3_C@RGO aerogel. The functional groups on the surface of the aerogel were identified by Fourier transform infrared spectrometer (FTIR, Bruker Vertex 70, Karlsruhe, Germany). The specific surface area and pore size distribution were analyzed by the Brunauer–Emmett–Teller method (BET, Quantachrome Quadrasorb SI, Boynton Beach, FL, USA). The chemical state of iron and the combination state of carbon in the aerogel were determined using an X-ray photoelectron spectrometer (XPS, Thermo Scientific K-Alpha, Waltham, MA, USA). The diffraction pattern was confirmed, and the ingredients of the aerogel were obtained from an X-ray diffractometer (XRD, Bruker D8 Advance, Karlsruhe, Germany). An electron paramagnetic resonance spectrometer (EPR, Bruker A300, Karlsruhe, Germany) was used to measure active radicals.

### 2.4. Catalytic Degradation Experimental of Methyl Orange by Self-Assembled Nano-Fe_3_C@RGO Aerogel

The methyl orange aqueous solution was prepared with a dilution of 12 mg/L. Hydrogen peroxide was dropped into the methyl orange solution until its concentration reached 160 mmol/L. Thereafter, 5 mg of the self-assembled nano-Fe_3_C@RGO aerogel was dipped into the solution and kept in a frozen water bath oscillator with an oscillated speed of 110 r/min for 2 h. During this reaction, a large number of active radicals were produced. Finally, the absorbance of the liquid supernatant was measured by a UV–vis spectrophotometer (UV759S, Shanghai INESA Scientific Instrument Co., Ltd., Shanghai, China) at a wavelength of 475 nm. The removal rate was calculated according to Formula (1).

(1) $$\eta =\frac{C_{0}-C_{t}}{C_{0}}$$

In the formula, η denotes the removal rate (%), $C_{0}$ and $C_{t}$ represent the concentration of the methyl orange solution before and after degradation, respectively (mg/L).

### 2.5. Analytical Methods

The generated active radicals were examined on a Bruker A300 EPR with 5,5-dimethyl-1-pyrroline *N*-oxide (DMPO) as the spin-trapping agent. The detection of hydroxyl radicals and superoxide radicals was conducted under the catalytic degradation experimental conditions described above.

## 3. Results and Discussion

### 3.1. The Morphology of Self-Assembled Nano-Fe_3_C@RGO Aerogel

**Figure 2:**
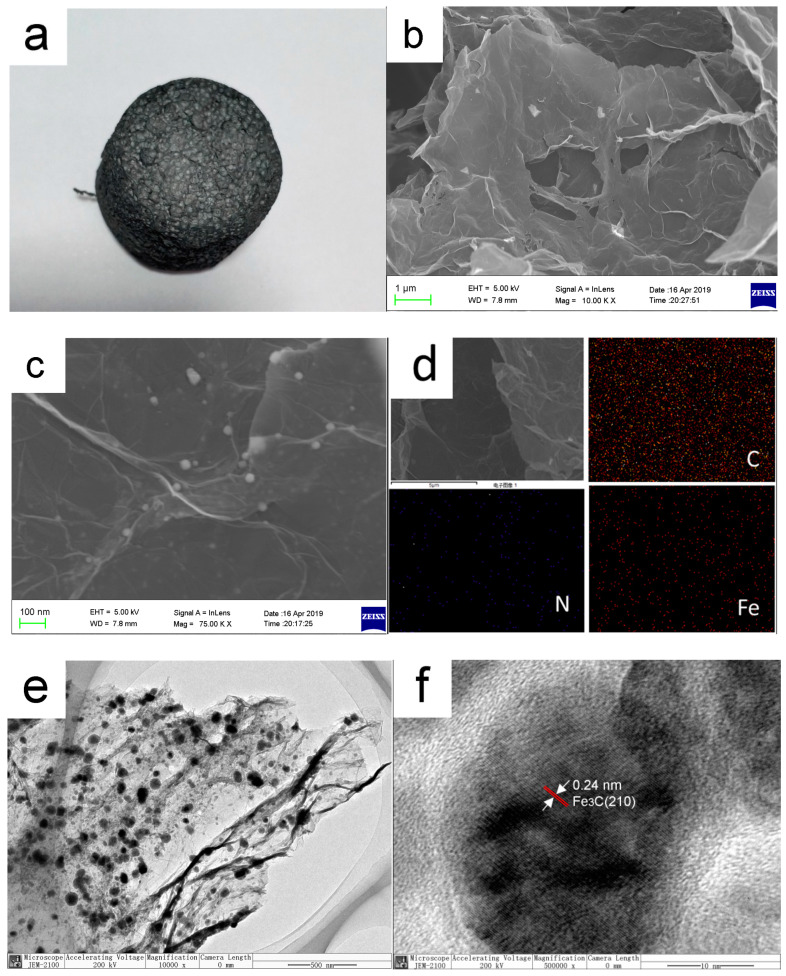
The morphology of self-assembled nano-Fe_3_C@RGO aerogel: (**a**) the macroscopic morphology; (**b**,**c**): SEM; (**d**) mapping; (**e**,**f**): HRTEM.

Figure 2a shows the macroscopic morphology of the self-assembled nano-Fe_3_C@RGO aerogel. It can be seen that the aerogel is well-formed and its color is gray and black. Figure 2b,c show the micromorphology of the aerogel. Figure 2b shows that the aerogel has a loose and porous structure and graphene is stacked layer by layer. Figure 2c reveals that its surface is doped with many evenly dispersed small particles which are nearly circular and less than 100 nm in diameter. In addition, most of the small particles are embedded in carbon and combined closely with graphene. Figure 2d makes it clear that the carbon, nitrogen and iron elements are homogeneously distributed in the aerogel, suggesting each part of the material has uniform catalytic properties. Figure 2e shows that the nano-Fe_3_C belongs to a uniform cladding structure. As seen in Figure 2f, the spacing of the central lattice fringes is ≈0.24 nm assigned to the (210) plane of the Fe_3_C phase, and the Fe_3_C nanoparticles are encapsulated in the graphitic carbon layers.

### 3.2. Pore Size Distribution

**Figure 3:**
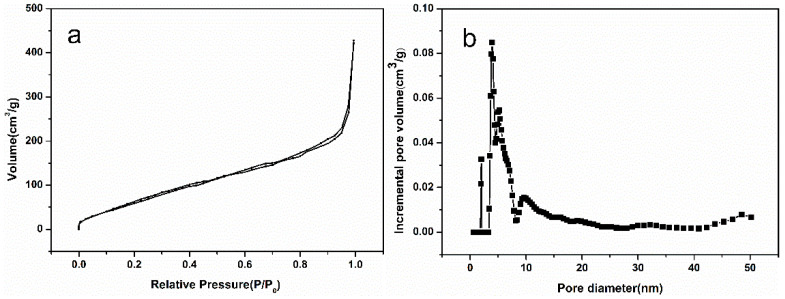
N_2_ adsorption-desorption isotherms and the pore size distribution: (**a**) N_2_ adsorption–desorption isotherms and (**b**) the pore size distribution.

Utilizing N_2_ adsorption–desorption isotherms, the microstructures of the obtained aerogel were further investigated, as shown in Figure 3. The hysteresis loop can be classified into H3 hysteresis effects, indicating that most of the pores inside the aerogel are mesoporous, which are wedge-shaped holes piled up by graphene sheets. According to Figure 3a, the BET specific surface area was calculated as 324.770 m^2^/g. As a rule, a higher specific surface area results in more active sites, which can be loaded with more catalysts. The pore size distribution curves calculated can be seen in Figure 3b. Most of the pore sizes are concentrated at 5–10 nm, which belong to a mesoporous structure. This pore size not only guarantees high specific surface area, but also provides a suitable channel for the entry and exit of organic molecules and H_2_O_2_, ensuring the catalytic reaction.

### 3.3. XRD

**Figure 4:**
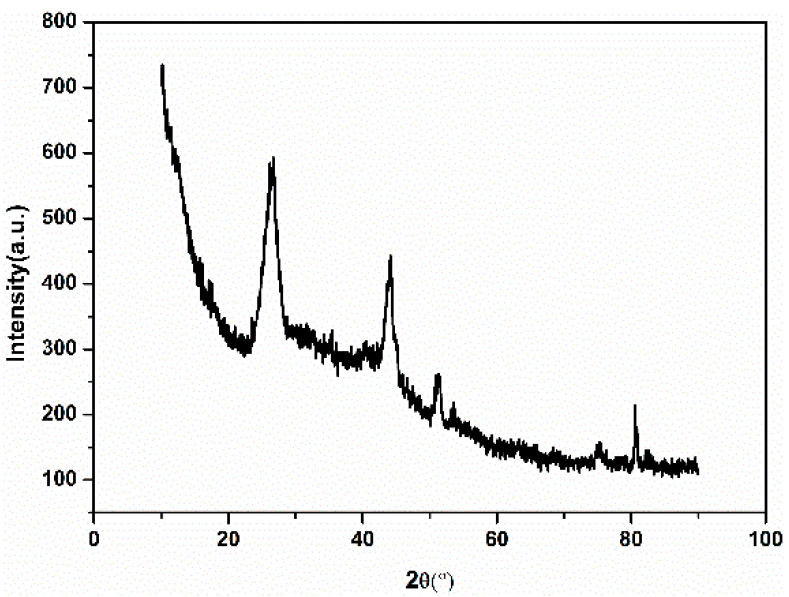
XRD of self-assembled nano-Fe_3_C@RGO aerogel.

A wide-angle XRD spectrum was further collected to characterize the self-assembled nano-Fe_3_C@RGO aerogel, as shown in Figure 4. The strong diffraction peaks at 26° could be indexed as graphite carbon. The other peaks at 35°, 44.5°, 51° and 81° (PDF 03-0411) with medium intensity could be indexed as Fe_3_C, which demonstrated the combination of Fe_3_C and RGO in the aerogel.

### 3.4. XPS

**Figure 5:**
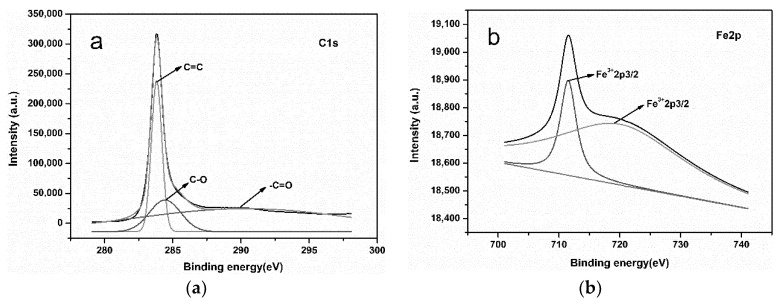
XPS of self-assembled nano-Fe_3_C@RGO aerogel: (**a**) C1s spectra and (**b**) Fe2p spectra.

To further confirm the carbon functional groups and iron valence state in the self-assembled nano-Fe_3_C@RGO aerogel, XPS was employed to analyze the aerogel. The C1s spectrum of the aerogel is shown in Figure 5a. Some functional groups are found and the three fitted peaks in this C1s XPS at around 284, 284.6 and 289 eV could be ascribed to C=C, C–O and –C=O, respectively. The peaks of C=C, C–O and –C=O were caused by RGO. The high resolution of the Fe2p spectrum in Figure 5b can be deconvoluted into a predominant peak at 711.5 eV (Fe^2+^ 2p_3/2_) and a satellite peak at around 719.3 eV due to the oxidation of Fe on the surface during preparation. The signal of zerovalent Fe, which is normally discovered in iron carbides (e.g., Fe_3_C) ≈ 707–708 eV, was not observed, further suggesting that the Fe_3_C nanoparticles are encapsulated by graphitic carbon layers [33], which is consistent with the HRTEM diagram in Figure 2f.

### 3.5. FTIR

**Figure 6:**
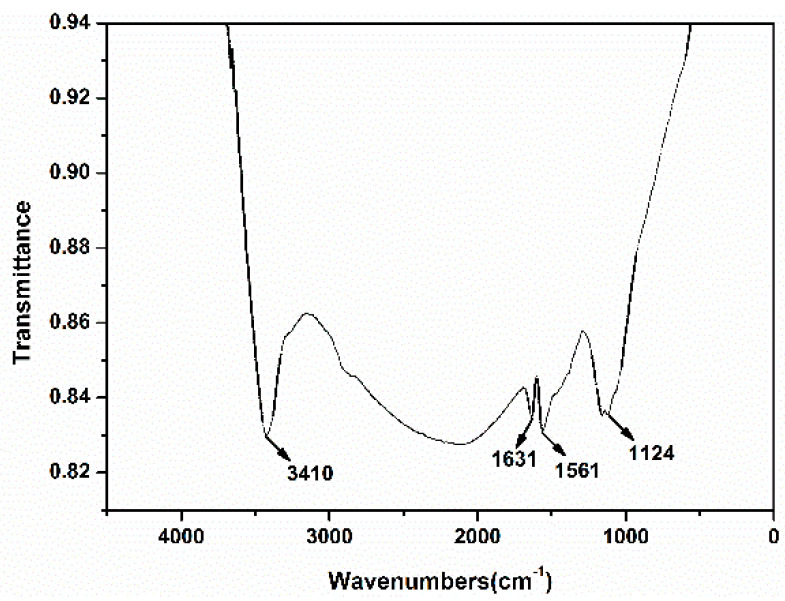
FTIR of self-assembled nano-Fe_3_C@RGO aerogel.

In order to understand the functional groups on the surface of the self-assembled nano-Fe_3_C@RGO aerogel, FTIR was characterized as shown in Figure 6. As can be seen from Figure 6, there is a strong peak at 3410 cm^−1^, which was mainly caused by the stretching vibration of –NH_2_ and –NH, possibly originating from the transformation of ammonia water at high temperature. The peak at 1561 cm^−1^ is due to the anti-symmetric stretching vibration of –NO_2_, which results from the transformation of the nitrate in the iron nitrate. The peak at 1631 cm^−1^ corresponds to the stretching vibration of C=C and –C=O of graphene, while the strong absorption peak at 1124 cm^−1^ is attributed to the stretching vibration of C–O. These results are consistent with those in Figure 5.

### 3.6. Catalytic Degradation Property of Self-Assembled Nano-Fe_3_C@RGO Aerogel for Methyl Orange

**Figure 7:**
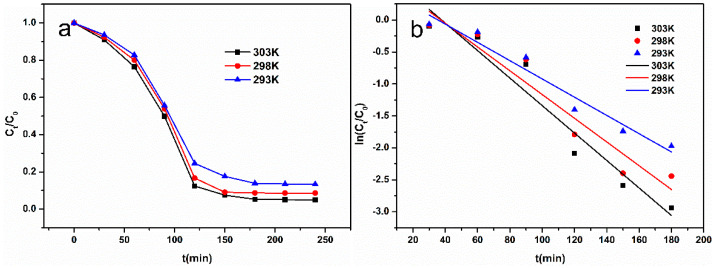

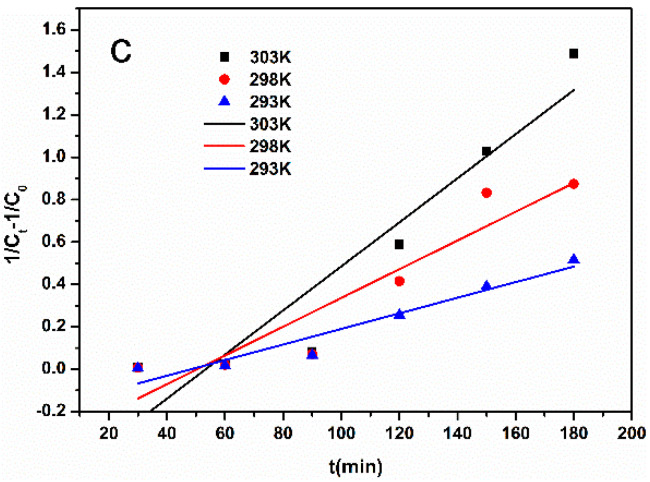
The catalytic degradation properties: (**a**) the kinetics of catalytic degradation; (**b**) the fitting diagram of first-order dynamics; (**c**) the fitting diagram of second-order dynamics.

In order to determine the reaction rate, the kinetics of catalytic degradation was investigated as shown in Figure 7a. At first, it can be seen that the value of *C_t_*/*C*_0_ decreased sharply and reduced to 0.075 at 303 K when the reaction time reached 150 min. Then, it decreased slowly to 0.050 when the reaction time was 240 min. This was mainly due to the high concentration of hydrogen peroxide, which could produce more hydroxyl radicals that could degrade more methyl orange. Thus, the reaction speed is faster at the beginning. In addition, the higher the temperature, the lower the equilibrium concentration, indicating that the reaction is more intense and complete at higher temperatures.

The first- and second-order kinetic equations are commonly used to describe adsorption degradation. The expressions of first-order dynamics equations are shown in Formula (2), and the fitting diagram of first-order dynamics is shown in Figure 7b. The fitting parameters are shown in Table 1.

(2) $$\ln\left(C_{t}/C_{0}\right)=-k_{1}t$$

In the formula, $C_{t}$ and $C_{0}$ represent the concentration of methyl orange after reaction time *t* and the initial solution, respectively (mg/L), and $k_{1}$ is the degradation rate constant of the first-order dynamics (min^−1^).

The second-order dynamics equation is expressed in Formula (3), and the fitting diagram of the second-order dynamics is drawn in Figure 7c. The fitting parameters are listed in Table 2.

(3) $$\frac{1}{C_{t}}-\frac{1}{C_{0}}=k_{2}t$$

In the formula, $C_{t}$ and $C_{0}$ represent the concentration of methyl orange at reaction time *t* and the initial solution (mg/L), respectively. $k_{2}$ is the degradation rate constant of the second-order dynamics (min^−1^).

**Figure 8:**
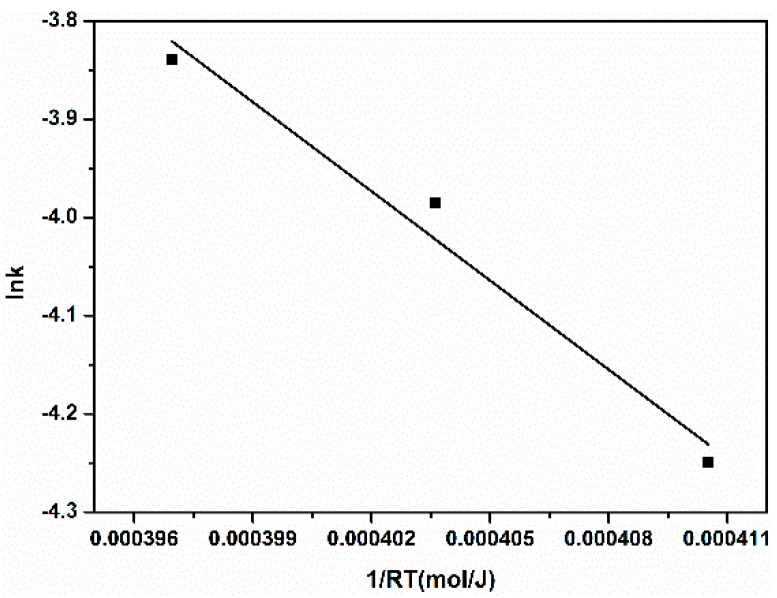
Arrhenius fitting plot of the catalysis degradation.

As seen as in Table 1 and Table 2, when the temperature is 293, 298 and 303 K, the correlation coefficients of the first-order kinetic model reach 0.9734, 0.9544 and 0.9634, respectively, which are larger than those of the second-order kinetic model. Therefore, the first-order kinetic model is more suitable for describing the catalytic degradation reaction rate and reaction process. According to Table 1, when the temperature is 293, 298 and 303 K, the degradation rate constants are 0.01428, 0.01858 and 0.0215 min^−1^, respectively, indicating that the reaction speed is accelerated with the increase in reaction temperature. This is because the higher temperature increases the catalytic capacity of Fe_3_C and accelerates the molecule movement of hydrogen peroxide and methyl orange, which is more suitable for catalytic degradation. The activation energy of chemical reactions is often estimated by the Arrhenius formula (Equation (4)). In order to calculate the activation energy of the catalytic degradation, the Arrhenius plot was fitted using to the temperature-dependent apparent rate constants shown in Figure 8. The fitting parameters are shown in Table 3.

Wu et al. state that when *E_a_* is between 8 and 21 kJ·mol^−1^, the reaction is a diffusion-controlled reaction, and when *E_a_* is >29 kJ·mol^−1^, the reaction is a surface-controlled reaction [34]. Therefore, the calculated *E_a_* of the catalysis degradation was 30.25 kJ·mol^−1^ (>29 kJ·mol^−1^), indicating that a surface-controlled reaction was occurring.

(4) $$\ln k=\ln A-\frac{E_{a}}{RT}$$

where *E_a_* (kJ·mol^−1^) is the activation energy; *A* (min^−1^) is a preexponential factor; *R* is equivalent to 8.314 J·(mol·K)^−1^; *T* is the temperature (K); *k* (min^−1^) is the reaction rate constant.

### 3.7. Catalytic Degradation Mechanism of Self-Assembled Nano-Fe_3_C@RGO Aerogel for Methyl Orange

**Figure 9:**
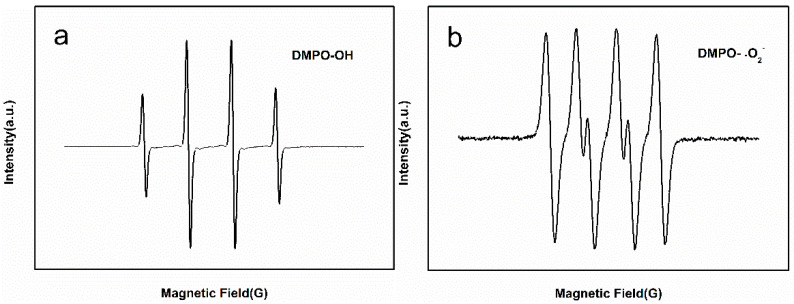
EPR spectra of: (**a**) DMPO-OH adducts and (**b**) DMPO-O_2_^−^ adducts.

**Figure 10:**
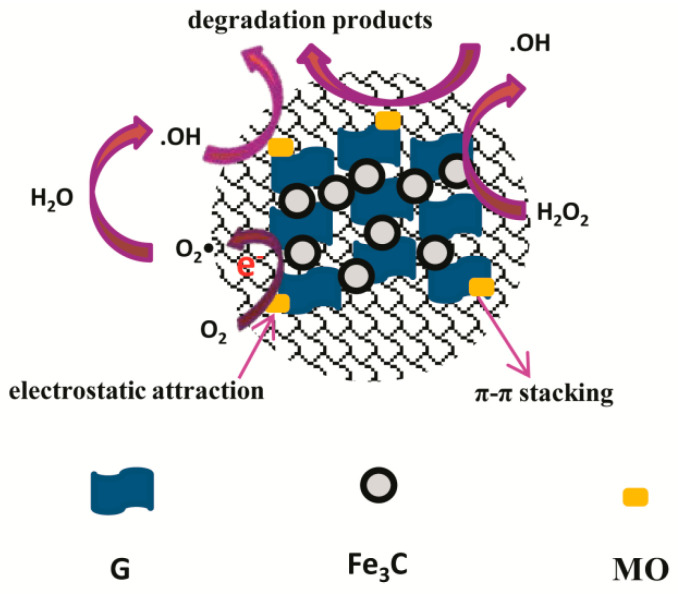
The catalytic degradation mechanism of methyl orange by self-assembled nano-Fe_3_C@RGO aerogel.

In order to make clear the catalytic degradation mechanism of the self-assembled nano-Fe_3_C@RGO aerogel for methyl orange, a radical quenching reaction was utilized to investigate the effect of radicals. The results are shown in Figure 9a,b, which indicated hydroxyl radicals [35] and superoxide radicals [36] can be generated in the reaction. According to the radical quenching experiments, the possible catalytic degradation mechanism of methyl orange by the self-assembled nano-Fe_3_C@RGO aerogel was proposed, as shown in Figure 10. First, methyl orange is adsorbed on the surface of graphene by electrostatic attraction, or π–π stacking. Then, hydrogen peroxide molecules diffuse to the surface of the graphene and contact Fe_3_C. Subsequently, hydrogen peroxide activated by Fe_3_C produces hydroxyl radicals. Moreover, some hydroxyl radicals are formed by superoxide radicals reacting with water [37]. Finally, methyl orange is oxidized with hydroxyl radicals on the surface of graphene and is degraded into small molecules.

## 4. Conclusions

A self-assembled nano-Fe_3_C@RGO aerogel was fabricated with hydrothermal synthesis and high-temperature treatment. It has a high specific surface area of 324.770 m^2^/g and abundant surface functional groups. Carbon, nitrogen, and iron elements were homogeneously distributed in the aerogel and Fe_3_C nanoparticles were encapsulated in the graphitic carbon layers. The self-assembled nano-Fe_3_C@RGO aerogel obtained excellent catalytic degradation for methyl orange under the synergistic effect of graphene and Fe_3_C. The first-order kinetic model can be used to describe the reaction rate and reaction process of catalytic degradation. Consequently, this aerogel is an ideal Fenton-like catalyst which can overcome the disadvantages of conventional Fenton reactions and has been applied to practical wastewater for the effective removal of organic pollutants.

## Figures and Tables

**Table 1:** First-order dynamic fitting parameters.

*T*	303 K	298 K	293 K
*k*_1_ (min^−1^)	0.0215	0.01858	0.01428
*r*	0.9634	0.9544	0.9734

**Table 2:** Second-order dynamic fitting parameters.

*T*	303 K	298 K	293 K
*k*_2_ (min^−1^)	0.00925	0.00528	0.00309
*r*	0.9148	0.8472	0.9190

**Table 3:** Arrhenius plot fitting parameters.

*E_a_* (kJ·mol^−1^)	ln*A*	*r*
30.25	8.1865	0.9761
